# Preservation Methods Differ in Fecal Microbiome Stability, Affecting Suitability for Field Studies

**DOI:** 10.1128/mSystems.00021-16

**Published:** 2016-05-03

**Authors:** Se Jin Song, Amnon Amir, Jessica L. Metcalf, Katherine R. Amato, Zhenjiang Zech Xu, Greg Humphrey, Rob Knight

**Affiliations:** a Department of Pediatrics, University of California—San Diego, La Jolla, California, USA; b Department of Ecology and Evolutionary Biology, University of Colorado—Boulder, Boulder, Colorado, USA; c Department of Anthropology, Northwestern University, Evanston, Illinois, USA; d Department of Computer Science and Engineering, University of California—San Diego, La Jolla, California, USA; University of Utah

**Keywords:** DNA stability, fecal microbiome, sample storage

## Abstract

Immediate freezing at −20°C or below has been considered the gold standard for microbiome preservation, yet this approach is not feasible for many field studies, ranging from anthropology to wildlife conservation. Here we tested five methods for preserving human and dog fecal specimens for periods of up to 8 weeks, including such types of variation as freeze-thaw cycles and the high temperature fluctuations often encountered under field conditions. We found that three of the methods—95% ethanol, FTA cards, and the OMNIgene Gut kit—can preserve samples sufficiently well at ambient temperatures such that differences at 8 weeks are comparable to differences among technical replicates. However, even the worst methods, including those with no fixative, were able to reveal microbiome differences between species at 8 weeks and between individuals after a week, allowing meta-analyses of samples collected using various methods when the effect of interest is expected to be larger than interindividual variation (although use of a single method within a study is strongly recommended to reduce batch effects). Encouragingly for FTA cards, the differences caused by this method are systematic and can be detrended. As in other studies, we strongly caution against the use of 70% ethanol. The results, spanning 15 individuals and over 1,200 samples, provide our most comprehensive view to date of storage effects on stool and provide a paradigm for the future studies of other sample types that will be required to provide a global view of microbial diversity and its interaction among humans, animals, and the environment.

**IMPORTANCE** Our study, spanning 15 individuals and over 1,200 samples, provides our most comprehensive view to date of storage and stabilization effects on stool. We tested five methods for preserving human and dog fecal specimens for periods of up to 8 weeks, including the types of variation often encountered under field conditions, such as freeze-thaw cycles and high temperature fluctuations. We show that several cost-effective methods provide excellent microbiome stability out to 8 weeks, opening up a range of field studies with humans and wildlife that would otherwise be cost-prohibitive.

## Abstract (precis)

Our study, spanning 15 individuals and over 1,200 samples, provides our most comprehensive view to date of storage and stabilization effects on stool. We tested five methods for preserving human and dog fecal specimens for periods of up to 8 weeks, including the types of variation often encountered under field conditions, such as freeze-thaw cycles and high temperature fluctuations. We show that several cost-effective methods provide excellent microbiome stability out to 8 weeks, opening up a range of field studies with humans and wildlife that would otherwise be cost-prohibitive.

## INTRODUCTION

Animals and their diverse microbial partners have coevolved for hundreds of millions of years, and microbes play a critical role in the health of many hosts. Studies of host-associated microbial communities have revealed important insights into the health, ecology, and evolution of various mammals (1–3), with most focusing on human health (4). For the study of microbiomes of the gastrointestinal tract, where most host-associated microbes reside, a critical aspect of sample acquisition is that feces can be sampled and used as a proxy for the lower intestinal (distal colon) microbiome. The recent surge of gut microbiome research has been made possible by this feature, coupled with technological advances in massively parallel sequencing platforms, advances in the cost of computation, and the development of powerful bioinformatic pipelines, allowing for large human microbiome initiatives such as the Human Microbiome Project (4) and the American Gut Project (http://www.americangut.org).

In many cases, including clinical settings, fecal samples can be collected via swabs or bulk collection and then immediately frozen at −20°C or below to prevent changes in the microbial community until DNA extraction can be completed. However, for samples collected in remote areas or requiring shipment, continuous access to a stable temperature at −20°C or lower may not be feasible. For example, accommodating immediate freezing of fecal material from humans living in remote hunter-gatherer communities (5) or of other organisms from remote regions of the world (6) can be challenging. Even in cases where a common household freezer is available, in studies where human participants are asked to self-sample and ship to a laboratory, for instance, samples may experience temperature fluctuations during storage (as home freezers undergo automatic defrost cycles) or during shipping, allowing samples to thaw. In regions with extreme temperatures or in cases of freezer failure, samples may additionally be exposed to extreme heat. In such instances, the use of an alternative stabilization method such as preservatives is necessary, but the effectiveness of commonly used preservatives is not well understood. Thus, quantification of the effects of these various methods of storage is critical to the expansion of microbiome studies beyond locations with access to freezers and electricity.

Previous studies have shown that the variations introduced by different preservation and storage methods usually do not outweigh differences between sample types (7), species (8), or individuals (9–11). However, these studies have generally been conducted over short time scales and using a limited sample size over a limited range of conditions. Microbiome studies are now quickly shifting toward identification of differences that are smaller in effect size, such as changes longitudinally within an individual, but with possibly large consequences for human, animal, and ecosystem health. Moreover, long-term biobanking of samples is becoming common practice, particularly for samples that are hard to obtain. Therefore, identification of effective storage methods that accurately and consistently capture these communities for extended periods of time is crucial.

**FIG 1:**
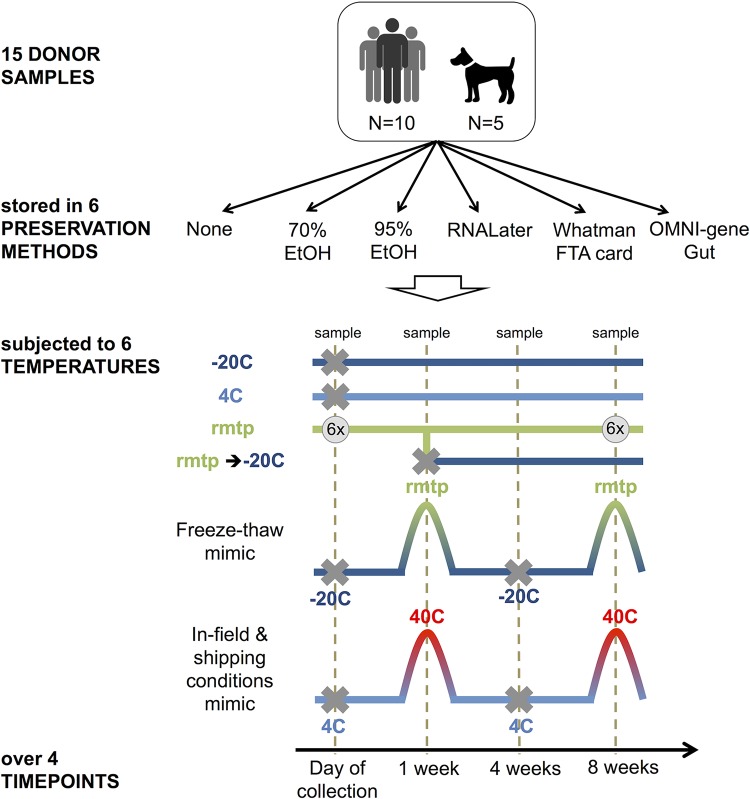
Diagram of experimental design. Bulk feces were collected from humans and dogs, homogenized, treated with a range of common preservatives, and subjected to a range of likely temperature scenarios. Conditions not sampled are marked with a gray X. Additional specific combinations of conditions not sampled are described in Materials and Methods and listed in Table S2 in the supplemental material. For freshly collected samples in no preservative, six replicate subsamples were taken from the same primary aliquot. At the 8-week time point, six replicate subsamples were taken from samples with no preservative and samples stored in 95% ethanol. rmtp, room temperature.

To better understand the effects of different storage preservatives and environmental conditions on the microbiota content of fecal samples as read out by 16S rRNA amplicon sequencing, we designed a laboratory experiment in which we collected fresh fecal samples from 10 human donors and 5 dog donors and tested the efficacies of four commonly used preservatives on the microbial community relative to both immediate extraction and the currently accepted standard of immediate freezing (Fig. 1). We tested ethanol (70% and 95% concentrations) and three commercially available reagents, RNAlater, OMNIgene Gut (DNA Genotek), and FTA cards (Whatman). We exposed each sample set to a variety of temperature conditions, which included constant temperatures of −20°C, 4°C, and ambient temperature, as well as temperature fluctuations mimicking freeze-thaw cycles and cyclic heat ramps (from 4 C to 40 C). We tested the effects of preservative type under each condition over an 8-week time period and extracted DNA at 4 time points: on the day of donation (“fresh”) and after 1, 4, and 8 weeks of storage. Specifically, we estimated bias introduced by preservation methods, which we define as differences in community composition in stabilized samples relative to fresh and immediately extracted samples (i.e., within 2 to 5 h of collection). We also compared samples stabilized through the use of a preservative against immediately frozen samples, which is currently considered the gold standard in sample storage and the state in which most biobanked samples are held. Furthermore, we estimated the stability of each preservation method, defined as microbial community changes over time relative to the baseline sample (i.e., sample extracted on the day of collection a few hours following exposure to a given preservative). Finally, we identified bacterial taxa that are likely to exhibit consistent decreases or increases in abundance under each type of storage condition, and thus may be useful indicators of fecal storage biases.

## RESULTS

### Storage effects are small compared to individual differences, even over 8 weeks.

**FIG 2:**
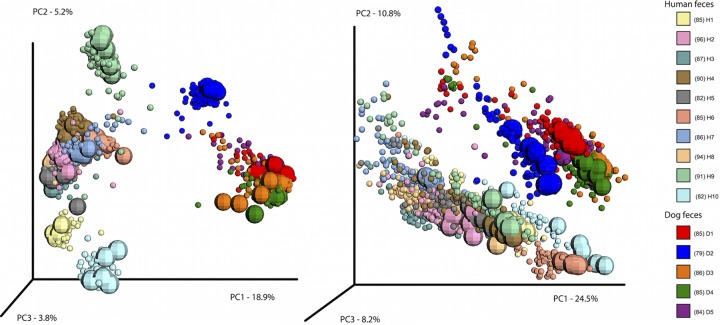
PCoA based on unweighted UniFrac (left) and weighted UniFrac (right) colored by individual with day 0 “fresh” samples (including replicates) represented with larger spheres. Bacterial communities of the 10 human participants are shown in pastel colors, and dogs are in brighter or darker colors.

It is well known that microbiome data sets vary in statistically detectable ways, even among technical replicates, such as multiple extractions of the same fecal samples. Therefore, we investigated the effects of different preservatives under different temperature regimes in the framework of relative effect size to contextualize the magnitude of the treatment effect. We compared differences among species (dog versus human), individuals (both humans and dogs), and among fresh sample replicates. This nested design allowed us to test the relative effects of these factors. A principal-coordinate analysis (PCoA) based on unweighted (presence/absence) and weighted (which accounts for relative abundance) UniFrac distances reveals that fecal microbial communities do change with treatments and conditions and that the community response to a stabilization method varies depending on donor, as treated samples scatter at various distances around the core group of replicate fresh samples (represented by the larger spheres in Fig. 2). However, the effect size is much smaller than variation between species, as humans and dogs are clearly separated along PC1 (Fig. 2). This was confirmed by a distance-based analysis of variability partitioning (adonis); interspecies and interindividual variability together explained 68% and 49% of the total variation in the data, as measured by weighted and unweighted UniFrac distances, respectively. The remaining variability was only minimally explained by preservation method (3% for weighted and 1% for unweighted), temperature (1% for weighted and 0.3% for unweighted), and storage time (0.7% for weighted and 0.001% for unweighted).

### Stabilization methods result in community shifts of various magnitudes compared to unstabilized fresh samples.

**FIG 3:**
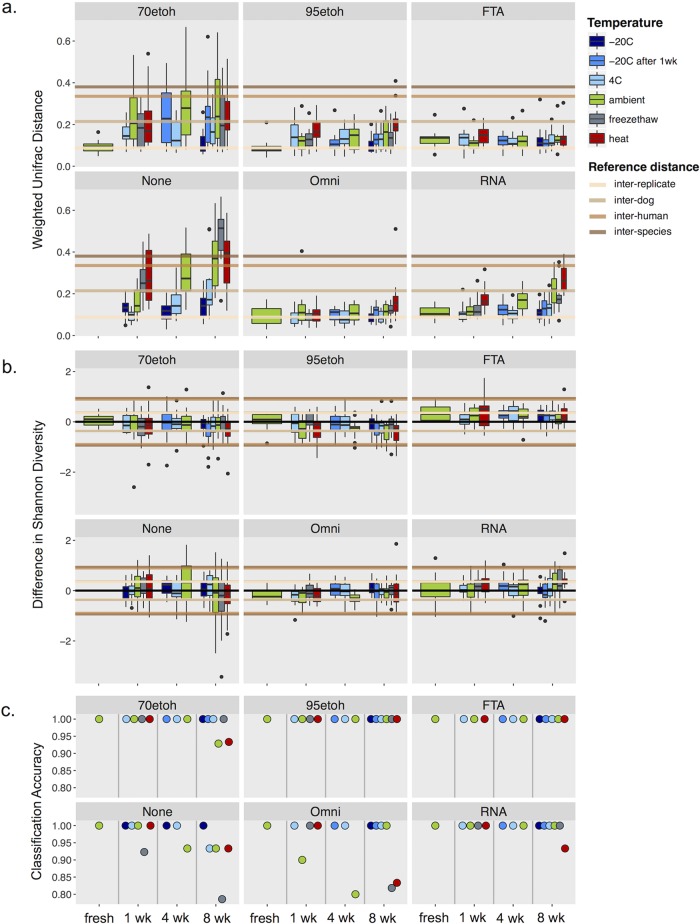
Summary of community changes by treatment over time. (a) Box plots show weighted UniFrac distances between treated samples and fresh samples taken on the day of sampling with no preservative. Horizontal lines show the average distance between replicate samples (lowest line, light tan), between different dogs (next line up, tan), between human individuals (next line up in dark tan), and between dogs and humans (highest line, brown). 70etoh and 95etoh, 70% and 95% ethanol, respectively. (b) Difference in alpha diversity is shown between samples indicated and the fresh sample. Reference absolute distances (interreplicate, interindividual, and interspecies) are shown in both the positive and negative directions. Note that the reference lines for interdog and interreplicate distances overlap significantly, as well as the lines for interhuman and interspecies distances. (c) The value shows the proportion of samples that were correctly assigned to the right individual using a random forest classifier. Colors represent the different temperatures at which samples were stored. “−20C after 1wk” represents samples that were stored at −20°C following 1 week of storage at ambient temperature.

We discovered that several stabilization methods prevented fecal microbial communities from changing in composition or diversity, even when exposed to temperature fluctuations. We found that immediately freezing a sample, particularly with the use of a preservative, causes the least change in composition and diversity, even after 8 weeks of storage (Fig. 3a and b; see Fig. S1 in the supplemental material). Fecal samples preserved in 95% ethanol, FTA cards, OMNIgene Gut, and RNAlater under most conditions had community compositional changes that were far smaller than the average difference between individual dogs or between individual humans (Fig. 3a; see Fig. S1). (Note that dogs are generally more similar to one another in gut microbiota than are humans.) In fact, both FTA cards and OMNIgene Gut resulted in fecal communities with compositional changes similar to those observed between technical replicates, suggesting that any changes introduced by these methods over time are within the limits of variation in the 16S rRNA amplicon sequencing assay. In contrast, unstabilized fecal samples and those preserved with 70% ethanol showed changes in microbial communities that were comparable in effect size to differences between individual humans and between species (Fig. 3a), except when samples were frozen or refrigerated at 4°C. (Note that we do not recommend the latter method because 4°C often encourages fungal growth, which is not addressed in this study but has often been dramatic in other samples we have received in our laboratory.) The size of the effect based on unweighted UniFrac distance is much smaller, suggesting that storage method affects the frequencies of the bacteria in a greater capacity than their absence/presence (Fig. S1; see Fig. S2 in the supplemental material). Further evidence of this is reflected in the alpha diversity estimates of the treated samples, as this was less dramatically affected by most treatments and preservative types, with average effect sizes similar to those observed between technical replicates (Fig. 3b). Unstabilized samples showed the largest amount of variation in alpha diversity changes, and interestingly, FTA cards consistently resulted in a slightly higher diversity of taxa under all conditions. To further investigate the magnitude of microbial community change caused by various temperature scenarios, we applied machine learning approaches, estimated the classification error for each sample to the correct individual, and discovered that the classification error was zero for fecal microbiomes preserved in 95% ethanol or with FTA cards (Fig. 3c). Misclassified samples tended to be those that were either left at room temperature for over a week or underwent temperature fluctuations (heat or freeze-thaw), although even under these conditions, misclassification rates were low.

10.1128/mSystems.00021-16.1 Figure S1  Box plots show unweighted UniFrac distances between treated samples and fresh samples taken on the day of sampling with no preservative. Horizontal lines show the average distances between replicate samples (lowest line in light tan), between different dogs (next line up in tan), between human individuals (next line up in dark tan), and between dogs and humans (highest line in brown). Colors represent the different temperatures at which samples were stored. “−20C after 1wk” represents samples that were stored at −20°C following 1 week of storage at ambient temperature. Download Figure S1, TIF file, 2.2 MB. Copyright © 2016 Song et al. 2016 Song et al. This content is distributed under the terms of the Creative Commons Attribution 4.0 International license.

10.1128/mSystems.00021-16.2 Figure S2  Box plots show weighted (upper panel) and unweighted (lower panel) UniFrac distances between treated samples and untreated samples frozen for 1 week. Horizontal lines show the average distances between replicate samples (lowest line in light tan), between dog individuals (next line up in tan), between human individuals (next line up in dark tan), and between dogs and humans (highest line in brown). Colors represent the different temperatures at which samples were stored “−20C after 1wk” represents samples that were stored at −20°C following 1 week of storage at ambient temperature. Download Figure S2, TIF file, 2.7 MB. Copyright © 2016 Song et al. 2016 Song et al. This content is distributed under the terms of the Creative Commons Attribution 4.0 International license.

### Stabilization methods vary in ability to minimize community changes over time.

**FIG 4:**
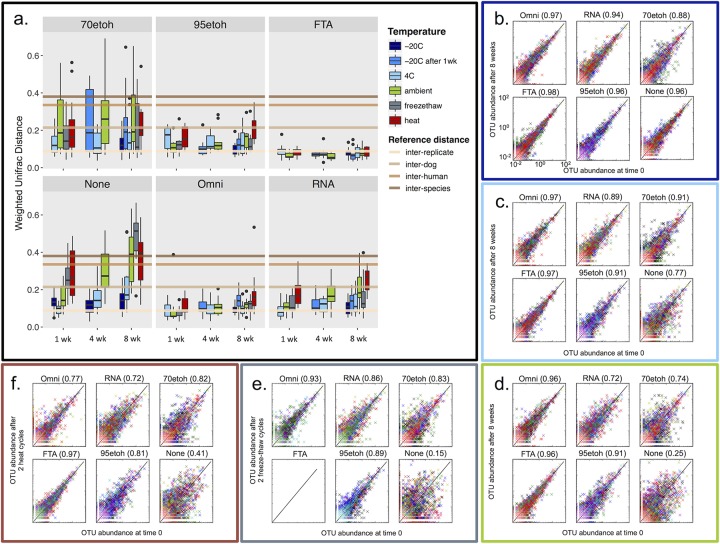
Stability of fecal microbiomes in different preservatives and under different temperature treatments. (a) Box plots show comparisons of weighted UniFrac distances between time points within each preservation type, within each individual, and across temperature treatments. Horizontal lines show the average difference in diversity between replicate samples (lowest line, light tan), between different dogs (next line up, tan), between human individuals (next line up in dark tan), and between dogs and humans (highest line, brown). Colors represent the different temperatures at which samples were stored. “−20C after 1wk” represents samples that were stored at −20°C following 1 week of storage at ambient temperature. 70etoh and 95etoh, 70% and 95% ethanol, respectively. (b to f) Scatterplots show the relative abundance (percentage) of each OTU found in the freshly collected sample placed in a given preservative, plotted against its relative abundance in the same preservative after 8 weeks, for each temperature manipulation, except in panel b, where the “None” plot shows the comparison between samples frozen for 1 week and samples frozen for 8 weeks. Colors correspond to different individuals. Correlation coefficients for each plot are shown in parentheses.

To test the stability of samples stored by each preservation method and to isolate the effect of preservation itself, we compared the microbial community composition and diversity of samples extracted on the day of collection immediately after adding a preservative to samples extracted after preservation for 8 weeks. We discovered that several methods resulted in very little change in microbial community composition (Fig. 4a) and diversity (Fig. 3b) over time. In particular, FTA cards and OMNIgene Gut had compositional changes similar to those observed between fresh technical replicates (Fig. 4a; FTA and Omni). RNAlater and 95% ethanol both showed slightly more compositional changes and appeared especially susceptible to change under heat conditions. Seventy percent ethanol performed poorly as a stabilizing condition for microbial communities, similar to using no preservative at all (Fig. 4a; “70etoh” and “None”). Without the additional use of a preservative, even immediately frozen samples showed compositional changes higher than what we observed between technical replicates. Relatedly, while samples preserved in 95% ethanol, FTA cards, OMNIgene Gut, and RNAlater were all very similar in composition to samples stored at −20°C for 1 week, weighted UniFrac distances were generally higher than those of fresh samples, also exceeding the average distance observed between technical replicates (see Fig. S2 in the supplemental material). We also compared changes in the abundance of operational taxonomic units (OTUs) that occurred after 8 weeks in a given preservative under each of the temperature conditions (Fig. 4b to e). At cold temperatures (−20°C and 4°C), we discovered very little change in relative abundance—as OTU showing no change in abundance will fall on the diagonal line—for fecal communities preserved on FTA cards and in OMNIgene Gut (correlation coefficient, *r* ≥ 0.97) and only slightly greater change for fecal samples preserved in 95% ethanol and RNAlater (*r* ≥ 0.91) (Fig. 4b and c). At ambient temperatures, however, RNAlater exhibited wider shifts in OTU abundance, dropping to a correlation coefficient of 0.72 (Fig. 4d). Under more extreme temperature conditions (freeze-thaw and heat), we observed a widening in the range of OTU abundance shifts for all preservatives except for FTA cards (Fig. 4e and f).

### Stabilization methods introduce biases in specific taxa.

**FIG 5:**
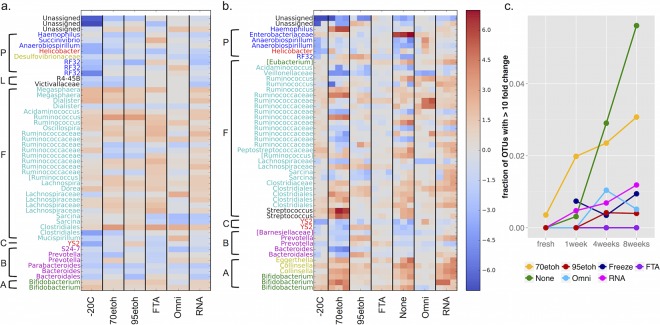
Distribution of the bacterial taxa that exhibit the greatest log fold changes in mean relative abundance. Heat maps show the log_2_ fold changes of the top 50 taxa that either increase (shown in red hues) or decrease (shown in blue hues) (a) once exposed to a preservation method (for 1 week at −20°C and a few hours in the other preservatives) and (b) over time within a preservation method (from left to right: 1, 4, and 8 weeks, except for −20°C, for which the 4- and 8-week time points are shown). Taxa are grouped by bacterial phyla (A, *Actinobacteria*; B, *Bacteroidetes*; C, *Cyanobacteria*; F, *Firmicutes*; L, *Lentisphaeria*; P, *Proteobacteria*) and colored by bacterial class. (c) A line graph shows the total fraction of OTUs that show a greater than 10-fold change in relative abundance for each stabilization method at each time point compared to the fresh sample. 70etoh and 95etoh, 70% and 95% ethanol, respectively.

We visualized the immediate and longer-term changes of microbial taxa (at the OTU level) observed once fecal samples underwent stabilization (Fig. 5). We should note that prior to running these analyses, we excluded one sample preserved with OMNIgene Gut, which exhibited characteristics indicative of technical errors or contamination because the community looked drastically different from all other samples from that individual, even those maintained in the buffer for longer periods. We observed small but immediate changes in the abundance of certain taxa when subjected to any stabilization method, including freezing, with OMNIgene Gut showing changes of the smallest magnitude in these taxa (Fig. 5a). Notably, in all stabilizers except OMNIgene Gut, most of the taxa that increased in relative abundance were *Firmicutes*, while taxa belonging to the phyla *Bacteroidetes* and *Proteobacteria* generally decreased. Changes in abundance occurring over time, however, varied across taxonomic groups, depending on which stabilizer was used, with FTA cards showing the smallest fluctuations in abundance of these taxa (Fig. 5b). Moreover, biases introduced by FTA cards appear to be sufficiently consistent that statistical corrections can be made (see Fig. S3 in the supplemental material). Using no preservative led to a significant increase in several OTUs, with the largest bloom occurring in an OTU belonging to the family *Enterobacteriaceae*. Using 70% ethanol led to similarly large increases in OTUs belonging to the bacterial genera *Streptococcus* and *Haemophilus*. By 8 weeks at ambient temperature, unstabilized samples and those stored in 70% ethanol showed the greatest number and proportion of OTUs undergoing dramatic changes in relative abundance. More specifically, nearly 6% of the taxa in no preservative and 3% of the taxa in 70% ethanol showed greater than a 10-fold change in relative abundance, compared to 1% in RNAlater and ≤0.5% in OMNIgene Gut or 95% ethanol and on FTA cards (Fig. 5c).

10.1128/mSystems.00021-16.3 Figure S3  Effect of preservative-based detrending of bacterial frequencies. Bray-Curtis distances are shown between untreated samples and samples in specific preservatives for each individual before and after frequency correction (*x* and *y* axes, respectively [see Materials and Methods]). The diagonal black line denotes no effect of correction. Correction results in FTA samples moving closer to untreated samples, while increasing the distance for other methods. Similar results were found for unweighted UniFrac distances after correction as well (not shown). Download Figure S3, TIF file, 2.8 MB. Copyright © 2016 Song et al. 2016 Song et al. This content is distributed under the terms of the Creative Commons Attribution 4.0 International license.

## DISCUSSION

Biological sampling in remote areas is very challenging, because samples must often endure weeks of transportation at various temperatures before they can be safely stored in a reliable lab-grade freezer. As studies of the microbiome expand in size, scale, and geographic breadth, the availability of alternative preservation methods is necessary to prevent changes in the microbial community as this is key for minimizing the potential for drawing erroneous conclusions. Moreover, quantification of the degrees of change associated with commonly used stabilizers can aid decisions in composing meta-analyses and making fair comparisons to biobanked samples that will be key for large initiatives, such as the Earth Microbiome Project, American Gut Project, and the Unified Microbiome Initiative. Our experiments reveal several preservative options useful in these and other field scenarios.

We have shown that fecal microbiomes can change substantially in composition over the course of 8 weeks in the absence of a stabilization method, especially when subjected to heat or freeze-thaw temperature fluctuations. Under less strenuous conditions, including a few days at room temperature without a fixative, shifts in the microbiome are of relatively small effect size compared to the variation between individuals. However, such differences can easily mask the detection of more subtle biological patterns of similar or smaller effect size and thus require much larger sample sizes. We show that these community shifts can be reliably minimized using several methods to a magnitude similar to that observed between sample replicates, specifically through the use of 95% ethanol, FTA cards, or OMNIgene Gut. Even in cases where immediate freezing is available, these options may deserve careful consideration as they each provided greater stability through the 8-week period under all temperature conditions. Perhaps most importantly, they provided increased protection from community changes from freeze-thaw cycles, which often occur during shipment, during sample processing, or if a sample undergoes multiple uses. RNAlater, on the other hand, while performing equally as well under most conditions, began losing stability at longer time frames when maintained at nonfreezing temperatures. According to the manufacturer’s instructions, this buffer, initially developed for the stabilization of RNA, is not designed to withstand ambient temperatures for longer than 2 weeks, as our data also suggest.

Due to the low cost and global accessibility of ethanol relative to other stabilizers, there has been much interest in the efficacy of ethanol for microbial community stabilization. However, previous studies have shown conflicting results, with reports of poor performance being associated with the use of a 70% solution (e.g., compare reference 12 versus 11), which has historically been acceptable for morphological preservation of biological specimens and for the preservation of macrofaunal DNA. However, even within the macrofaunal community, it has been recognized that DNA is best preserved in 95 to 100% ethanol as higher concentrations allow for more rapid penetration of cellular membranes and deactivation of DNases (13). Our data also suggest that ethanol is indeed a viable option for long-term storage of fecal samples for microbiome analysis at concentrations of 95% or higher. Some studies have identified low DNA yield as one potential drawback of using ethanol, resulting in lower concentrations compared to frozen or RNAlater-preserved samples (12, 14). Consistent with these findings, ethanol-preserved samples (both 70% and 95%) tended to yield, on average, the lowest concentrations of DNA among all of the methods we tested in our study as well (results not shown), despite preserving the DNA with high reliability. However, as we did not explicitly quantify or standardize the volume of fecal material input into each preservative type, we cannot state with confidence that the variation in DNA yield can be attributed to certain preservation or storage methods.

FTA or equivalently FOBT (i.e., fecal occult blood test) cards have been shown by several studies to be good stabilizers of DNA (11). One major advantage of these cards is their ability to remain stable for remarkably long periods of time at room temperature. However, also consistent with other studies, we found that FTA cards tend to recover a greater diversity of bacterial taxa than other preservation methods (12). Contamination stemming from the cards themselves is unlikely to be the cause of this increase, given that 12 out of the 15 sample-free FTA cards we extracted alongside our samples failed to yield measurable amounts of DNA, failed PCR, and resulted in fewer than 100 sequencing reads. One possible explanation may be that chemical lysing through the FTA card matrix may allow detection of additional taxa than through the extraction process alone. Extraction from a fresh sample may not necessarily reflect the true community, because detection relies on a combination of factors, including cell lysis and extraction efficacy. Many taxa showing increased abundance in FTA card-preserved samples belong to the phylum *Firmicutes*, which are often sporeformers, providing some evidence that more efficient lysis may be the cause. Yet if this were the case, we may also expect freezing to lead to a similar increase in diversity of detected taxa due to its effects on cell wall structure. Freezing has in fact been shown to result in a higher ratio of *Firmicutes* to *Bacteroidetes*, presumably due to the ability of Gram-positive bacteria to preserve greater DNA stability than Gram-negative bacteria through the freezing process (15, 16). In a recent study, Dominianni et al. (9) also reported that freezing led to a more diverse recovered community than from RNAlater-preserved or fresh samples. While we did observe changes that resulted in a different recovered community from the fresh sample, freezing did not lead to the recovery of an increased diversity of taxa in our study. Future studies using mock communities consisting of bacteria of defined taxonomy and growth stages may help to resolve this issue, although results from pure cultures will not necessarily match those from specimens in a complex matrix, such as stool or soil.

As a relatively new product, OMNIgene Gut has not yet faced the same levels of scrutiny as other commercially available stabilizers, although a growing number of studies now support it as a strong contender for use in microbiome studies. One recent study found it to perform better than RNAlater and Tris-EDTA at maintaining the composition of fecal microbial community structure (17), in concordance with the results of our study. We have some data based on agarose gel banding that suggest OMNIgene Gut may also yield greater DNA quality than other methods (results not shown); however, more robust qualitative and quantitative testing of all of these methods is needed to better evaluate their effectiveness at preserving optimal DNA quality and yield.

### Conclusions.

Several methods now exist for storing samples stably at room temperature for weeks, allowing long-term field trials.

We recommend the use of 95% ethanol, OMNIgene Gut, or FTA cards for long-term studies and strongly caution against the use of 70% ethanol for any period or unfixed samples over more than a few days. Any of the three recommended methods will result in differences due to storage that are smaller than or comparable to differences among technical replicates, although more sensitive methods, such as shotgun metagenomics or quantitative PCR (qPCR), may reveal differences not observed in this study.

Many additional considerations we do not discuss here will likely contribute to a researcher’s choice of stabilization method, including cost, availability, ease of use, processing time and effort, whether special shipping for flammable liquids is required, and whether the preservative is compatible with other “omic” methods. For example, many research groups are beginning to integrate the investigation of multiple molecules such as DNA, RNA, and metabolites concurrently from the same sample. Hence, for some studies, using a fixative that is suitable for multiple types of molecules (e.g., 95% ethanol) may outweigh the benefits of using one optimized for a single molecule type. However, the present results demonstrate that a wide range of field-collected specimens are suitable for microbiome analysis and that much cheaper methods can be used for field collections than have previously been deployed.

## MATERIALS AND METHODS

### Sample collection.

Ten humans and five dogs each donated ~20 g of fresh stool. The human volunteers gave consent under the University of Colorado, Boulder, IRB protocol 0409.13. The 5 dog samples were obtained under the approved IACUC protocol from the University of Colorado, 1401.01. These samples were homogenized in a plastic bag and immediately aliquoted into or onto each of the six treatment types (no preservative, 70% ethanol, 95% ethanol, Whatman FTA card, RNAlater, or OMNIgene Gut) per the manufacturer’s instructions, where provided, such that an aliquot of each type could be extracted at four different time points after storage at ambient temperature (~21 C): on the day of collection (fresh) and at 1, 4, and 8 weeks. Samples stored at 4°C and −20°C were extracted after 1, 4, and 8 weeks of storage. Subaliquots of the samples stored at ambient temperatures were transferred to −20°C after 1 week, where they remained until extraction following an additional 3 and 7 weeks of storage. Samples that underwent freeze-thaw and heat cycles were sampled at 1 week immediately following one temperature fluctuation cycle and at 8 weeks following two cycles (Fig. 1). FTA cards were not subjected to freeze-thaw cycles because the manufacturer’s descriptions state stability at room temperature for indefinite periods of time. FTA card- and OMNIgene Gut-preserved samples were prepared for DNA extraction following the manufacturer’s instructions. For all other preservatives, sterile cotton swabs (Puritan Medical Products, Guilford, ME) were used to subsample feces prior to extraction. This resulted in a total of 1,230 samples, not including 109 extraction blanks, 15 FTA controls (blank FTA cards), and 180 technical replicates, which consisted of five additional subsamples of the same aliquot.

### 16S amplicon sequencing and processing.

For each sample, we extracted DNA and generated 16S rRNA amplicons from fecal samples using Earth Microbiome Project standard protocols (http://www.earthmicrobiome.org/emp-standard-protocols/). Briefly, at each of the time points shown in Fig. 1, fecal samples from each treatment and temperature manipulation were extracted using the MoBio Powersoil kit (96-well plate). We amplified the 16S rRNA V4 region with golay error-correcting bar codes on the 515f forward primer so that sequences could be demultiplexed after sequencing. Our pool of 16S rRNA amplicons were sequenced on both an Illumina Hiseq 2500 in rapid run mode and an Illumina MiSeq at the University of California—San Diego, Institute for Genomic Medicine. Sequence data from the HiSeq run were supplemented with reads from MiSeq for 19 samples which failed PCR and failed to sequence on HiSeq but successfully sequenced on the MiSeq; these are listed in Table S1 in the supplemental material. These substitutions did not significantly affect our results as removal of these samples resulted in similar conclusions (data not shown). The forward read sequences from the HiSeq and MiSeq platforms, which were 95 to 125 nucleotides (nt) and 150 nt in length, respectively, were trimmed to 125 nt, quality filtered, and demultiplexed using QIIME version 1.9.0 (18). These reads were clustered and assigned to OTUs with 97% similarity to the Greengenes 13.8 reference database using the sortmerna algorithm. Remaining reads were clustered *de novo* using sumaclust, and taxonomy was assigned using uclust. A phylogenetic tree was built using all OTUs, which was used to calculate phylogeny-informed distances between samples (e.g., UniFrac distances). We rarefied to 10,000 sequences per sample, resulting in the retention of 1,127 samples (see Table S2 in the supplemental material), plus 171 technical replicates.

10.1128/mSystems.00021-16.4 Table S1  List of samples that failed to sequence on the Illumina HiSeq and for which MiSeq reads were substituted. Download Table S1, PDF file, 0.03 MB. Copyright © 2016 Song et al. 2016 Song et al. This content is distributed under the terms of the Creative Commons Attribution 4.0 International license.

10.1128/mSystems.00021-16.5 Table S2  Breakdown of the number of samples that were analyzed for each of the preservation methods at each time point and temperature condition. Download Table S2, PDF file, 0.04 MB. Copyright © 2016 Song et al. 2016 Song et al. This content is distributed under the terms of the Creative Commons Attribution 4.0 International license.

### Diversity measures.

We estimated the compositional similarities between microbial communities (beta diversity) using both unweighted and weighted UniFrac metrics (19, 20), which estimate distance between samples based on the shared branch lengths (i.e., phylogenetic distances) among the microbial lineages in those communities. We used the resulting distance matrix to generate a principal-coordinate ordination plot. We compared the effect sizes of preservation and temperature treatments to the effect sizes by individual and by species by using a distance-based approach to variation partitioning using adonis in the vegan R package (21) with 999 permutations. We estimated the microbial diversity represented within each fecal sample using the Shannon diversity index.

### Bias and stability.

We analyzed the effects of preservation on bacterial composition and diversity in two ways: (i) bias, or the effect of using a stabilization method compared to a nonstabilized fresh sample; and (ii) stability, or the change over time in a sample compared to the initial time point of the sample using a given stabilization method. All analyses were done on a within-individual basis and then summarized across individuals. Bias introduced through freezing as a stabilizing method was tested by comparing the fresh sample to one that was stored at −20°C for 1 week. Biases introduced through the use of preservatives were tested by comparing a fresh sample exposed briefly to the respective preservative (~2 to 5 h at room temperature prior to extraction) to a nonstabilized freshly extracted sample. To determine which method was most comparable to freezing, we compared the communities of preserved samples to those that had been frozen for 1 week.

### Classification of individuals.

In order to assess how well the community composition retained signatures of an individual with treatment over time, we used a supervised learning approach to train our models on fresh replicate samples (6 per individual) and estimated classification accuracy for all of the other conditions. The classification model was built using a random forest method and tuned with cross-validation using the caret R package (22).

### Fold change and taxon-specific analyses.

OTU fold changes between samples were calculated as the difference in relative abundance of an OTU in a sample compared to another sample. Only OTUs found in both samples above a minimum abundance of 0.05% were considered in this analysis. Significance of composition difference between samples was calculated using permutation tests. Briefly, the mean OTU fold change values between the two treatments were compared to values obtained after permutation of the methods (for each OTU across all individuals).

### Detrending.

For each preservation method, preservative-treated samples were randomly split into training (12/15 of subjects) and validation (3/15 of subjects) sets. For each OTU, the mean fold change between time 0 preservative-treated and time 0 untreated samples was calculated on the training set (averaged over all subjects where the OTU is present). This per-OTU fold change value was then used to correct the observed frequency in the validation set, and the average Bray-Curtis distance to the time 0 untreated sample of the same subject was calculated. The whole process was iterated 50 times. Bray-Curtis distances were used to evaluate detrending performance because this metric is not influenced by whether changes in the frequencies are in closely or distantly related OTU, but rather treats all OTU equally.

### Accession numbers.

Sequence data and associated metadata are available in Qiita under study ID no. 10394 (https://qiita.ucsd.edu/study/description/10394) and in the European Bioinformatics Institute European Nucleotide Archive (accession number ERP015155).
