# Supplementary figures and images for: Preservation Methods Differ in Fecal Microbiome Stability, Affecting Suitability for Field Studies
Source: mSystems. 2016 May 3;1(3):e00021-16. doi: 10.1128/mSystems.00021-16 (PMC5069758; doi:10.1128/mSystems.00021-16)

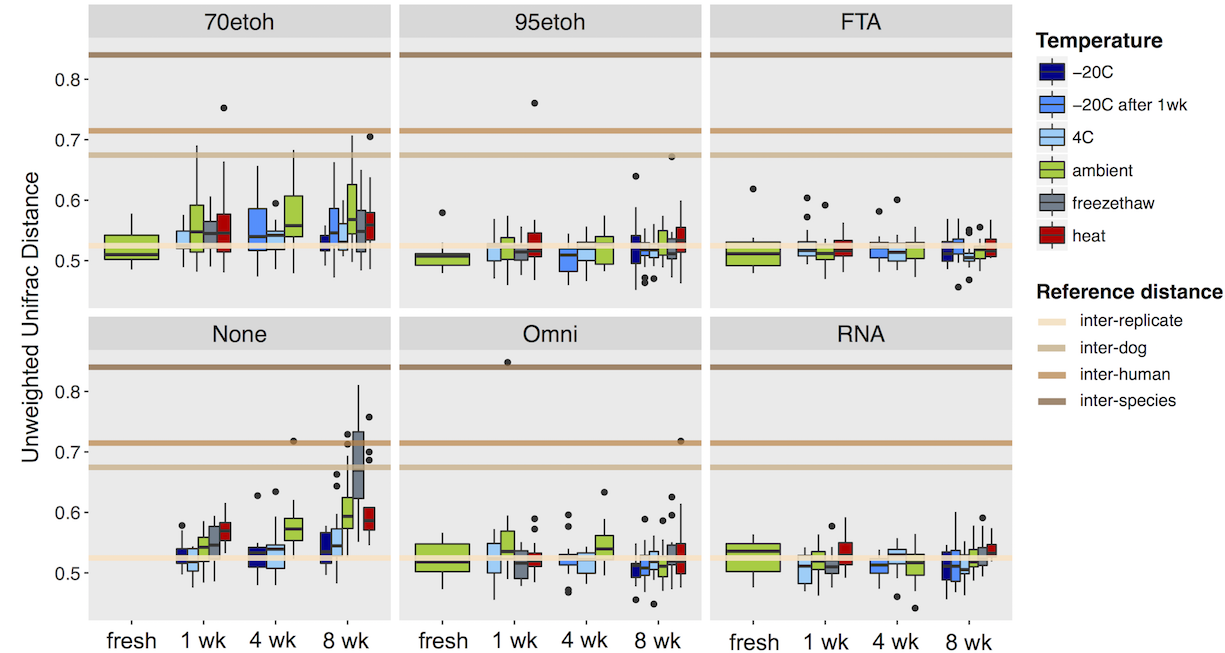

Supplement: Figure S1 [file sys001162019sf1.tif]

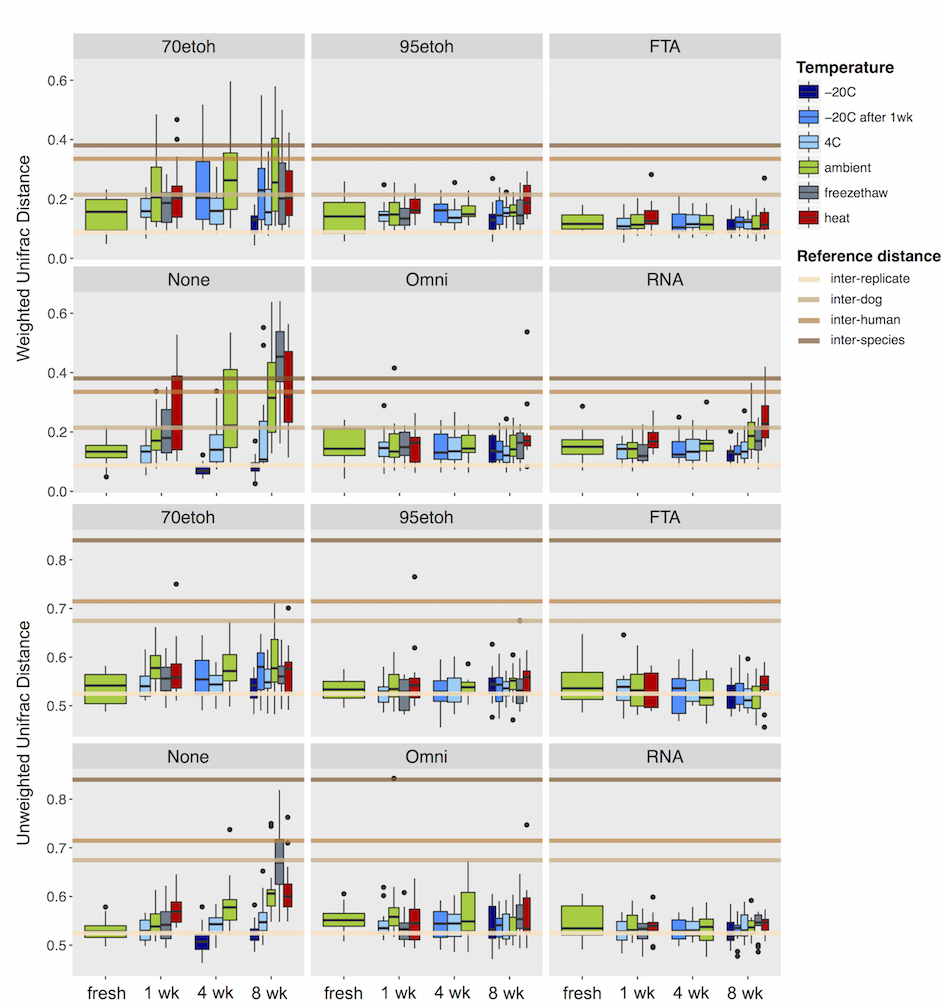

Supplement: Figure S2 [file sys001162019sf2.tif]

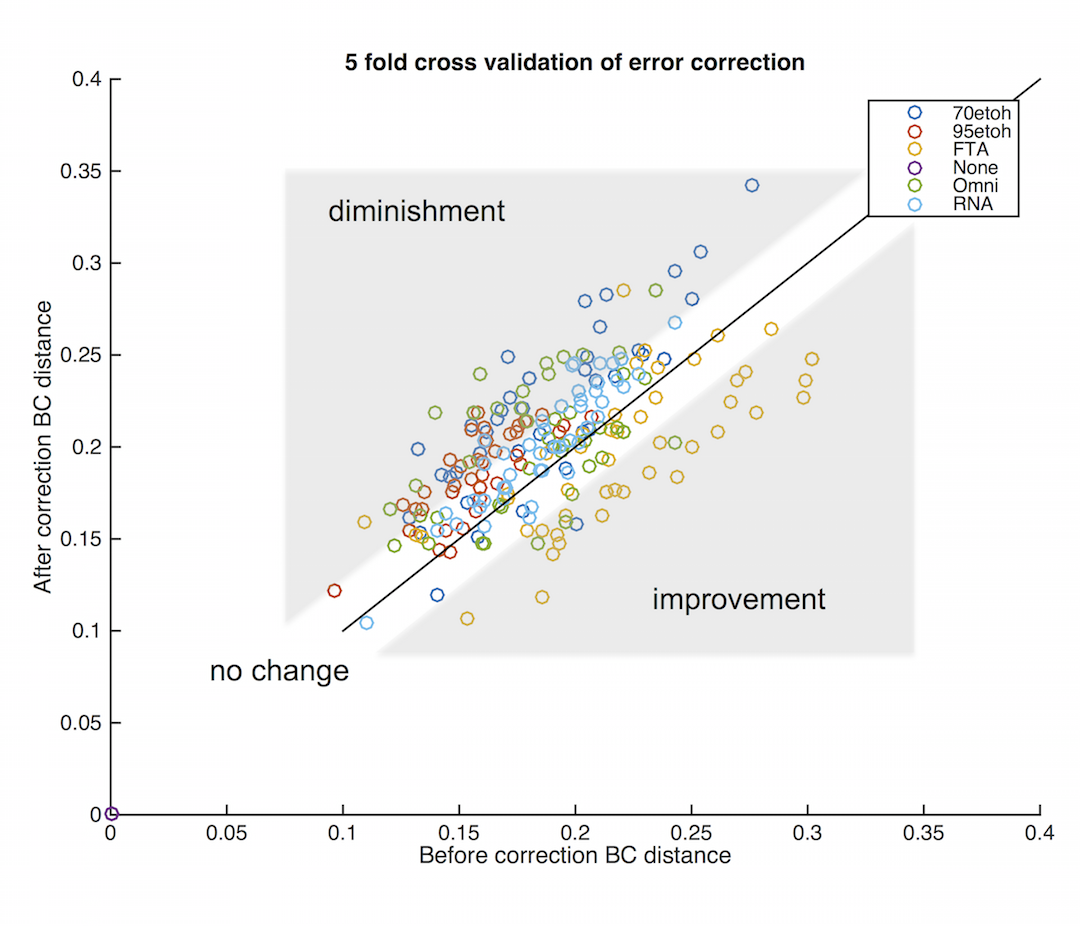

Supplement: Figure S3 [file sys001162019sf3.tif]
