# Supplementary material for: Preservation Methods Differ in Fecal Microbiome Stability, Affecting Suitability for Field Studies
Source: mSystems. 2016 May 3;1(3):e00021-16. doi: 10.1128/mSystems.00021-16 (PMC5069758; doi:10.1128/mSystems.00021-16)
Supplement: Table S1 [file sys001162019st4.pdf]

Sample name
D1.FTA.heat.1week.II
D2.70etoh.amb.1week.II
D2.FTA.heat.1week.II
D2.rep1.amb.fresh.II
D4.rep5.amb.fresh.II
D5.70etoh.amb.fresh.II
H1.rep4.amb.fresh.II
H2.70etoh.amb.fresh.II
H2.RNA.amb.fresh.II
H3.None.amb.fresh.II
H3.rep4.amb.fresh.II
H5.rep4.amb.fresh.II
H5.RNA.4C.1week.II
H6.FTA.heat.1week.II
H7.FTA.heat.1week.II
H7.rep4.amb.fresh.II
H8.FTA.heat.1week.II
H9.FTA.heat.1week.II
H10.FTA.heat.1week.II
