# Supplementary material for: Preservation Methods Differ in Fecal Microbiome Stability, Affecting Suitability for Field Studies
Source: mSystems. 2016 May 3;1(3):e00021-16. doi: 10.1128/mSystems.00021-16 (PMC5069758; doi:10.1128/mSystems.00021-16)
Supplement: Table S2 [file sys001162019st5.pdf]

preservative	temperature	timepoint				total
		fresh	1week	4weeks	8weeks	
None	-20C		15	14	15	44
	-20C after 1 wk					
	4C		15	15	15	45
	amb	15	15	15	15	60
	freezethaw		13		14	27
	heat		14		15	29
70etoh	-20C				12	12
	-20C after 1 wk			14	15	29
	4C		13	15	14	42
	amb	12	15	14	14	55
	freezethaw		14		14	28
	heat		15		15	30
95etoh	-20C				15	15
	-20C after 1 wk			13	15	28
	4C		13	13	15	41
	amb	10	14	13	15	52
	freezethaw		13		13	26
	heat		12		14	26
FTA	-20C				13	13
	-20C after 1 wk			12	14	26
	4C		15	14	13	42
	amb	11	14	15	15	55
	freezethaw					
	heat		14		13	27
OMNIgene Gut	-20C				15	15
	-20C after 1 wk			15	12	27
	4C		14	14	11	39
	amb	12	10	10	15	47
	freezethaw		13		11	24
	heat		14		12	26
RNAlater	-20C				13	13
	-20C after 1 wk			13	15	28
	4C		14	14	15	43
	amb	13	15	15	13	56
	freezethaw		15		13	28
	heat		14		15	29
